# Prospective randomized study on the efficacy of tamsulosin, solifenacin, and their combination in relieving lower urinary tract symptoms in ureteric stent patients: insights from the brief-form Chinese USSQ

**DOI:** 10.1007/s00345-025-05695-1

**Published:** 2025-05-16

**Authors:** Peter Hanna, Mohamed A. Fahmy, Mohamed M. Hussein, Amr Alam El-din

**Affiliations:** Department of Urology, Aswan University, Aswan, Egypt

**Keywords:** Solifenacin, Tamsulosin, Upper ureter symptoms score, Ureteral stent

## Abstract

**Purpose:**

Assess the efficacy of selective alpha-1 blocker (tamsulosin) and antimuscarinic (solifenacin) as well as their combination in alleviating lower urinary tract symptoms in patients with ureteric stents using the brief-form Chinese version of the USSQ.

**Methods:**

A prospective randomized control study was conducted from May 2023 to October 2024 in the Urology department at Aswan University Hospital involving 160 consecutive patients undergoing DJ insertion after uncomplicated ureteroscopy. All patients are randomized into 4 groups receiving different treatments. Groups A, B, C, and D received a placebo, tamsulosin 0.4 mg, solifenacin 5 mg, and combinations respectively. IPSS and USSQ (brief-version) were collected and analyzed on the 1st day, 1st week, and 3rd week postoperative.

**Results:**

In all, 158 patients completed the study, 39 patients for Group A and Group B, while 40 patients for Group C and Group D. Baseline characteristics were comparable among all groups. Group (D) showed a significant decrease in mean ± (SD) IPSS 9.5 (± 1.4), and 7.7 (± 1.3) on 1st week and 3rd week postoperative respectively. Regarding USSQ, group (D) showed significantly lower scores compared to groups (A), (B), and (C) on the 1st day, 1st week, and 3rd week postoperatively (p < 0.001, p < 0.001, p < 0.001; respectively).

**Conclusion:**

The brief-form USSQ is a useful, reliable substitute for evaluating stent-related symptoms. Our findings support the notion that tamsulosin/solifenacin combinations have considerably reduced LUTS linked to the placement of ureteral stents.

**Supplementary Information:**

The online version contains supplementary material available at 10.1007/s00345-025-05695-1.

## Introduction

Ureteral stents were initially introduced by Zimskind et al. in 1967 [1, 2]. Following continuous improvement, particularly in the double-pigtailed design [3], the ureteral stent has emerged as an essential urological instrument, facilitating the relief of both acute and chronic urethral obstruction through dilation and urine drainage [4].

A recent systematic review indicated that the omission of stents after ureteroscopy was linked to a higher chance of unexpected medical visits and readmissions [5]. Nonetheless, individuals with an indwelling ureteral stent, irrespective of duration, might experience a range of stent-related symptoms (SRSs), such as frequency (50–60%), urgency (57–60%), dysuria (40%), incomplete emptying (76%) flank pain (19–32%), suprapubic pain (30%), incontinence and hematuria (25%) [6], impacting the quality of life in around 80% of patients [7].

To cut down the rate of stent-related symptoms (SRSs), early attempts have been undertaken to enhance the physical characteristics of ureteral stents, including the material, length, shape, and placement. Nonetheless, given that the variety of stent sizes and designs aimed at reducing SRSs appears restricted, the ideal stent has yet to be created [8]. Nonetheless, oral medication has demonstrated positive effects, with alpha-blockers and anti-muscarinics being the most commonly used [2, 9].

Despite being nonspecific, the International Prostate Symptom Score (IPSS) was commonly utilized to evaluate SRSs. Subsequently, Joshi et al. recognized patient morbidity linked to ureteric stents as a major health issue; they created an easy-to-use and validated tool to more accurately assess the adverse effects on patients’ QoL named the Ureteric Stent Symptom Questionnaire (USSQ) [10]. The USSQ consists of 38 items examining six sections: pain, voiding symptoms, work performance, sexual matters, overall general health, and additional problems [11].

An eight-item symptom score questionnaire adapted from previously validated work and published literature is a brief-form Chinese version of the USSQ that was widely applied for evaluation of stent-related symptoms, which encompass the severity of (urinary frequency, urgency, urge incontinence, nocturia), (flank pain, abdominal pain, urethral pain, and hematuria). Each item was offered a score from (0 to 5). The individual index has a score ranging from 0 to 5, with high scores corresponding to worse outcomes [12, 13].

We sought in our study to compare the overall effectiveness of tamsulosin versus solifenacin versus their combinations in controlling symptoms associated with stent insertion using the brief-form Chinese version of the USSQ.

## Patients and methods

### Study population

This study is a prospective randomized control study conducted from May 2023 to October 2024 in the Urology department at Aswan University Hospital, Egypt to assess the efficacy of tamsulosin vs. sofenacin vs. combination for treatment of the symptoms associated with DJ insertion. All patients aged from 18 to 45 years old were included in our study. Exclusion criteria include patients older than 45 years, diabetic patients, patients with a history of stent hypersensitivity, pregnant women, preoperative anticholinergic or alpha-blockers, bilateral DJ, and migrated JJ stents or neglected JJ stents (103 patients; supplementary Table 1). Of total 160 consecutive patients were conducted in our study. All patients had DJ insertion after uncomplicated semirigid ureteroscopy and stone extraction. With a diameter of 6 Fr and lengths of 24 and 26 cm, the ureteral stent was made of polyurethane. The patient’s height determined the ureteral stent’s length. The patients are randomized into 4 groups receiving different treatment options using sealed envelopes (placebo, tamsulosin 0.4 mg, solifenacin 5 mg, or combinations).

### Outcomes

To evaluate the effectiveness of tamsulosin vs. solifenacin vs. their combinations for alleviating symptoms caused by DJ-stent placement following uncomplicated ureteroscopy.

The Ureteral Symptom Score Questionnaire (USSQ) was a validated and frequently used questionnaire for symptoms related to stents. Lower urinary tract symptoms were assessed using the brief-form Chinese USSQ, which was translated into Arabic using a rigorous forward and back-translation process to ensure semantic equivalence with the original English and Chinese versions. It encompasses urinary symptoms score and body pain score, the two primary components of USSQ (supplementary Table 2).

The initial part of the questionnaire consisted of four questions aimed at evaluating the severity of urinary frequency, urgency, urgent incontinence, and nocturia, which were derived from the validated International Prostate Symptoms Score (IPSS) questionnaire. The questionnaire’s second part consisted of four items aimed at evaluating the intensity of body flank pain, abdominal pain, urethral pain, and hematuria scored from 0 to 5, where 0 indicates no pain and 5 signifies the most severe pain experienced.

### Sample size

To determine the necessary participant numbers for this study, an a priori sample size calculation was conducted using G*Power software (Version 3.1.9.4; Faul et al., 2007) [14]. The input parameters were based on findings from a previous trial [15]. Based on an anticipated medium to large effect size (Cohen’s f = 0.4), a high statistical power of 0.95, and a conventional significance level of α = 0.05, the analysis yielded a required total sample size of 112 participants. To account for potential attrition, a 25% dropout rate was factored in, increasing the estimated total sample size to 140 (112 + 28). This adjusted sample size was subsequently divided equally among the four study groups, resulting in a target of at least 35 participants per group (supplementary Fig. 1).

### Statistical analysis

All comparisons made between continuous variables were conducted using the ANOVA test for parametric data or the Kruskal–Wallis test for non-parametric data. Then, pairwise comparisons were conducted utilizing Dunn’s (1964) method along with a Bonferroni adjustment for multiple comparisons. P-values have been adjusted and are shown. Comparisons are made among categorical variables with the Chi-square test or Fisher Exact test, followed by post hoc analysis that includes pairwise comparisons and utilizes the z-test of two proportions alongside a Bonferroni correction. All statistical analyses were carried out using SPSS v 25 (IBM SPSS Statistics for Windows, Version 25.0. Armonk, NY: IBM Corp.).

## Results

Of total 160 patients, 2 patients were excluded from the final analysis due to lost follow-up. Finally, 39 patients remained in the placebo group (Group A) and tamsulosin group (Group B), however, 40 patients were in both the solifenacin group (Group C) and combinations group (Group D). Overall, 158 patients were analyzed.

Baseline characteristics of all groups are listed in Table 1. There were no significant changes between groups regarding age, gender, BMI, stone site, or side. Also, there were no significant differences among groups regarding baseline IPSS.

**Table 1:** Patients’ demographics and baseline characteristics

	Group (A) N = 39	Group (B) N = 39	Group (C) N = 40	Group (D) N = 40	P value
Age (years)					0.82
Mean (± SD)	32.3 (6.5)	31.8 (7.1)	30.7 (5.8)	32.6 (7.4)	
Gender N (%)					0.91
Male	28 (71.8%)	29 (74.4%)	28 (70%)	30 (75%)	
Female	11 (28.2%)	10 (25.6%)	12 (30%)	10 (25%)	
BMI					0.74
Mean (± SD)	20.4 (3.6)	19.4 (2.6)	21.7 (3.5)	22.7 (3.8)	
Stone site N (%)					0.83
Upper	10 (25.6%)	12 (30.8%)	8 (20%)	8 (20%)	
Middle	15 (38.5%)	15 (38.5%)	20 (50%)	22 (55%)	
lower	14 (35.9%)	12 (30.8%)	12 (30%)	10 (25%)	
Side N (%)					0.97
Right side	19 (48.7%)	21 (53.8%)	15 (37.5%)	17 (42.5%)	
Left side	20 (51.3%)	18 (42.6%)	25 (62.5%)	23 (57.5%)	

On the 1 st day postoperative, mean ± (SD) IPSS was 15.1 ± (2.6), 14.2 ± (3.5), 14.4 ± (1.8), and 11.5 ± (1.3) for group (A), group (B), group (C), and group (D) respectively (supplementary Table 3). On the 1 st week postoperative, the mean ± (SD) IPSS of the group (D) 9.5 (± 1.4) was significantly decreased compared to other groups. Also, on the 3rd week postoperative, the mean ± (SD) IPSS of the group (D) 7.7 (± 1.3) was significantly lower than the other 3 groups (supplementary Fig. 1).

On pairwise comparison, for the 1 st day postoperative (POP), group (D) shows a significant decrease in IPSS when compared to group (A), group (B), and group (C) by 3.564, 2.553, 2.550 respectively (p < 0.001, p < 0.001, p < 0.001; respectively). Similarly, group (D) shows a significant decrease in IPSS in 1 st week (p < 0.001, p = 0.022, p < 0.001; respectively) and 3rd week postoperatively (p < 0.001, p < 0.001, p < 0.001; respectively) compared to other 3 groups (Table 2).

**Table 2:** Pairwise comparison of postoperative IPSS among all groups. P-values in bold are significant

	Groups	1 st postoperative day		1 st postoperative week		3rd postoperative week	
		Mean differ	Significance*	Mean differ	Significance*	Mean differ	Significance*
Placebo	Tamsulosin	0.942	0.402	2.436	** < 0.001**	3.308	** < 0.001**
	Sofenacin	1.002	0.387	1.150	**0.034**	3.405	** < 0.001**
	Combination	3.564	** < 0.001**	3.475	** < 0.001**	5.505	** < 0.001**
Tamsulosin	Placebo	− 0.942	0.402	− 2.436	** < 0.001**	− 3.308	** < 0.001**
	Sofenacin	0.033	0.983	− 1.286	0.062	1.003	0.843
	Combination	2.553	** < 0.001**	1.039	**0.022**	2.197	** < 0.001**
Sofenacin	Placebo	− 1.002	0.387	− 1.150	**0.034**	− 3.405	** < 0.001**
	Tamsulosin	− 0.033	0.983	1.286	0.062	− 1.003	0.843
	Combination	2.550	** < 0.001**	2.325	** < 0.001**	2.100	** < 0.001**
Combination	Placebo	− 3.564	** < 0.001**	− 3.475	** < 0.001**	− 5.505	** < 0.001**
	Tamsulosin	− 2.553	** < 0.001**	− 1.039	**0.022**	− 2.197	** < 0.001**
	Sofenacin	− 2.550	** < 0.001**	− 2.325	** < 0.001**	− 2.100	** < 0.001**

The Double J-related symptom scores (brief-form USSQ) are listed in supplementary Table 2. On the 1 st day postoperatively, the (mean ± SD) of the total score was significantly lower in group (D) 13.97 ± 1.96 compared to group (A), group (B), and group (C) 21.77 ± 1.98, 17.94 ± 1.94, and 16.97 ± 2.19 respectively (p < 0.001, p < 0.001, p < 0.001; respectively). Similarly, group (D) showed a significantly lower (mean ± SD) score of 14.67 ± 1.78, and 14.77 ± 1.43 in 1 st week and 3rd week postoperatively respectively (p < 0.001, p < 0.001; respectively) (Table 3).

**Table 3:** Pairwise comparisons of double-J stent-related symptoms score (brief-from USSQ) among different treatment groups on 1 st day, 1 st week and 3rd week postoperatively. P-values in bold are significant

	Groups	Mean ± SD (total score)	Mean diff	Confidence interval (CI)		P value*
				Lower	Upper	
	1 st day postoperative					
Tamsulosin	Placebo	21.77 ± 1.98	− 3.76	− 5.00	− 2.53	** < 0.001**
	Solifenacin	16.97 ± 2.19	.97	−.25	2.19	0.212
	Combination	13.97 ± 1.96	3.97	2.74	5.19	** < 0.001**
Solifenacin	Placebo	21.77 ± 1.98	− 4.74	− 5.96	− 3.51	** < 0.001**
	Tamsulosin	17.94 ± 1.94	−.97	− 2.19	.25	0.212
	Combination	13.97 ± 1.96	3.00	1.78	4.21	** < 0.001**
Combination	Placebo	21.77 ± 1.98	− 7.74	− 8.96	− 6.51	** < 0.001**
	Tamsulosin	17.94 ± 1.94	− 3.97	− 5.19	− 2.74	** < 0.001**
	Solifenacin	16.97 ± 2.19	− 3.00	− 4.21	− 1.78	** < 0.001**
	1 st week postoperative					
Tamsulosin	Placebo	22.54 ± 2.40	− 4.51	− 5.86	− 3.16	** < 0.001**
	Solifenacin	16.15 ± 1.93	.055	− 1.28	1.39	0.98
	Combination	14.67 ± 1.78	2.55	1.21	3.89	** < 0.001**
Solifenacin	Placebo	22.54 ± 2.40	− 4.56	− 5.90	− 3.22	** < 0.001**
	Tamsulosin	17.23 ± 2.68	−.055	− 1.39	1.28	0.98
	Combination	14.67 ± 1.78	2.50	1.16	3.83	** < 0.001**
Combination	Placebo	22.54 ± 2.40	− 7.06	− 8.40	− 5.72	** < 0.001**
	Tamsulosin	17.23 ± 2.68	− 2.55	− 3.89	− 1.21	** < 0.001**
	Solifenacin	16.15 ± 1.93	− 2.50	− 3.83	− 1.16	** < 0.001**
	3rd week postoperative					
Tamsulosin	Placebo	21.54 ± 3.01	− 3.01	− 4.58	− 1.43	** < 0.001**
	Solifenacin	19.10 ± 2.23	− 0.57	− 2.13	0.99	0.92
	Combination	14.77 ± 1.43	3.75	2.18	5.31	** < 0.001**
Solifenacin	Placebo	21.54 ± 3.01	− 2.43	− 3.99	− 0.88	** < 0.001**
	Tamsulosin	18.52 ± 2.52	0.57	− 0.99	2.13	0.92
	Combination	14.77 ± 1.43	4.32	2.78	5.86	** < 0.001**
Combination	Placebo	21.54 ± 3.01	− 6.76	− 8.31	− 5.20	** < 0.001**
	Tamsulosin	18.52 ± 2.52	− 3.75	− 5.31	− 2.18	** < 0.001**
	Solifenacin	19.10 ± 2.23	− 4.32	− 5.86	− 2.78	** < 0.001**

The treatment regimens, including tamsulosin, solifenacin, and their combination, demonstrated good tolerability. The occurrence of adverse events was minimal, infrequent, and did not necessitate medication discontinuation in any participant.

## Discussion

Ureteral stents, despite ensuring urinary drainage, commonly induce bothersome lower urinary tract symptoms (LUTS), significantly impairing patients’ quality of life [16]. The precise mechanism contributing to discomfort linked to ureteric stents remains unclear; however, several authors have noted that this discomfort is related to ureteric spasms, urinary reflux caused by the stent, or trigonal irritation [17].

This is the first prospective randomized controlled study comparing 3 different treatment options (alpha-blocker vs. antimuscarinic vs. their combination) to alleviate symptoms related to stent insertion using an updated brief Chinese version of the Ureteral Symptom Score Questionnaire (Ch-USSQ).

The underlying mechanisms of stent-related symptoms are still not well understood. A potential cause for stent-associated LUTS was the spasm of the bladder and lower ureter caused by irritation of the intravesical segment of the stent [18]. The involuntary contractions of the bladder were facilitated by muscarinic receptors [19]. Likewise, the discomfort, spasm, and elevated pressure transmitted to the renal collecting system through the stent may explain the pain experienced in the body (urethra, abdomen, and flank) [20]. While gross hematuria has been assumed as closely linked to stent friction in the collecting system caused by physical activity and reduced urine volume, severe and persistent spasms along with retrograde pressure transmission through the stent are also associated with hematuria [21].

In our study, IPSS was significantly decreased in group (D) on 1 st day, 1 st week, and 3rd week postoperative compared to group (A), group (B), and group (C) (p < 0.001, p < 0.001, p < 0.001; respectively).

In the same context, Lim et al. [22] conducted a retrospective study involving 168 patients categorized into 4 groups based on treatment received after DJ insertion. The initial group received no treatment (Group 1), 43 patients took tamsulosin 0.2 mg daily (Group 2), 45 patients were administered solifenacin 5 mg daily (Group 3), and 32 patients were provided with a combination of two drugs after surgery. The study showed a significant reduction in total IPSS in the combination group when compared to tamsulosin (p = 0.013).

In our study, group (B) and group (C) showed significant improvements in USS compared to placebo postoperatively. Yet, they showed comparable results to each other. Group (D) showed a significant decrease in USS in comparison to the other 3 groups on 1 st day, 1 st week, and 3rd week postoperatively respectively.

A randomized control trial aligned with our results showed that group IV patients who received combination therapy showed a statistically significant difference in all scores as compared to groups of patients receiving tamsulosin and solifenacin, respectively (*P* value < 0.001) [23].

On the other hand, a randomized controlled trial was conducted with 112 patients who underwent unilateral ureteral stent insertion following ureteroscopic stone surgery. The patients were randomly assigned into 4 groups A (control), B (tamsulosin 0.2 mg once daily), C (solifenacin 5 mg once daily), and D (both active treatments). It showed that urinary symptom scores at week 2 and week 6 were not significantly different between the four groups (*p* = 0.323 and *p* = 0.430, respectively). In the same way, the four groups’ scores on the five USSQ domains in week two were comparable [15].

The different results reported by the previous study may be attributed to the higher age group included in the study (mean ± SD, 54 years ± 11.3). Moreover, differences in stent lengths among groups of study participants make the results inaccurate. Lastly, a modest sample size with a higher dropout rate is a strong limitation to generalize those results.

Our study has some limitations. First, we didn’t compare between groups regarding the side effects of medications used. Second, the USSQ is a six-domain, validated questionnaire that is frequently used to evaluate symptoms linked to stents. As a result, we used the Chinese brief-form USSQ translated into Arabic to evaluate the symptoms associated with the stent. The Chinese version, brief-form USSQ has not been evaluated to prove its validity and reliability in the Chinese community, even though back translation was done to ensure that the translation includes every component of the original version. We conducted a prospective randomized control study using this brief-form USSQ to assess its credibility in comparison between tamsulosin, solifenacin, and their combinations in mitigation of LUTS associated with ureteral stent insertion. Validation of such brief-form USSQ in further studies can save time on follow-up visits in urology clinics resulting in mitigating medical, nursing, and financial burdens.

## Conclusion

Brief-form USSQ could be a valuable credible alternative tool to assess the symptoms related to stents. Our results add to the evidence that the tamsulosin/solifenacin combination has significantly alleviated LUTS associated with ureteral stent insertion.

## Supplementary Information

Below is the link to the electronic supplementary material.

Supplementary file1 (DOCX 239 KB)

## Data Availability

No datasets were generated or analysed during the current study.
