# Supplementary material for: Prospective randomized study on the efficacy of tamsulosin, solifenacin, and their combination in relieving lower urinary tract symptoms in ureteric stent patients: insights from the brief-form Chinese USSQ
Source: World J Urol. 2025 May 16;43(1):313. doi: 10.1007/s00345-025-05695-1 (PMC12084230; doi:10.1007/s00345-025-05695-1)
Supplement: Supplementary file 1 — Supplementary file1 (DOCX 239 KB) [file 345_2025_5695_MOESM1_ESM.docx]

**Supplementary material**

**Figures**


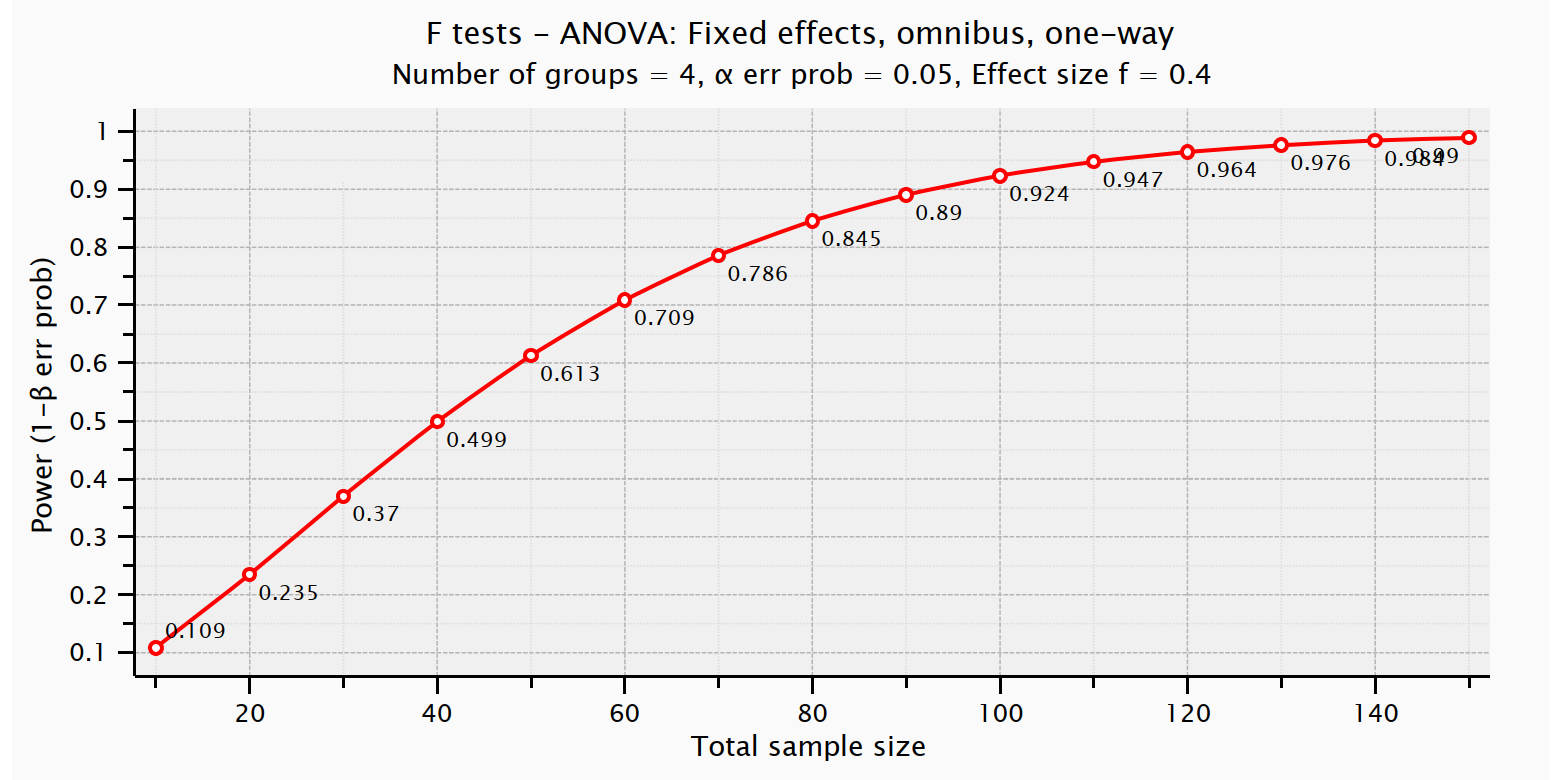


**Figure (1): Plot of estimated sample size using G*power program**

**Figure (2):** Plots of means of IPSS among 4 groups on 1^st^ day, 1^st^ week and 3^rd^ week postoperatively.

| **Exclusion Criterion** | **Number of Patients Excluded** |
| --- | --- |
| Older than 45 years | 22 |
| Diabetic patients | 17 |
| Past history of stent hypersensitivity | 3 |
| Pregnant women | 8 |
| Preoperative anticholinergic use | 13 |
| Preoperative alpha-blocker use | 20 |
| Bilateral DJ stents | 13 |
| Migrated JJ stents | 0 |
| Neglected JJ stents | 7 |
| Total Excluded | 103 |

**Table (1):** Exclusion criteria with corresponding patients’ numbers

**Table (2)**: the brief-form Chinese version of the Ureteral Stent Symptom Questionnaire (USSQ)

| 1. How often have you had to urinate less than 2 hours again after you finished urination?  (0) Not at all  (1) Less than one time in 5  (2) Less than half the time  (3) About half the time  (4) More than half the time  (5) Almost always |
| --- |
| 2. How often have you found it difficult to postpone urination?  (0) Not at all  (1) Less than one time in 5  (2) Less than half the time  (3) About half the time  (4) More than half the time  (5) Almost always |
| 3. How often have you had a urine leak before you can get to the toilet?  (0) Not at all  (1) Less than one time in 5  (2) Less than half the time  (3) About half the time  (4) More than half the time  (5) Almost always |
| 4. How many times did you most typically get up to urinate from the time you went to bed until the time you got up in the morning?  (0) Never  (1) 1 time  (2) 2 times  (3) 3 times  (4) 4 times  (5) 5 times or more |
| 5. Have you ever had any flank pain?  Try to grade the severity on a scale of 0 to 5 (with 0 being no pain and 5 being the worst pain ever experienced)  (0)  (1)  (2)  (3)  (4)  (5) |
| 6. Have you ever had any abdominal pain?  Try to grade the severity on a scale of 0 to 5 (with 0 being no pain and 5 being the worst pain ever experienced)  (0)  (1)  (2)  (3)  (4)  (5) |
| 7. Have you ever had any urethral pain?  Try to grade the severity on a scale of 0 to 5 (with 0 being no pain and 5 being the worst pain ever experienced)  (0)  (1)  (2)  (3)  (4)  (5) |
| 8. How often have you ever had hematuria?  (0) Not at all  (1) Less than one time in 5  (2) Less than half the time  (3) About half the time  (4) More than half the time  (5) Almost always |

**Table (3): comparisons of preoperative and postoperative IPSS among groups**

|  | **Group (A)**  **N=39** | **Group (B)**  **N=39** | **Group (C)**  **N=40** | **Group (D)**  **N=40** | **P value**** |
| --- | --- | --- | --- | --- | --- |
| Baseline IPSS  Mean (±SD) | 3.4 (± 0.8) | 2 (± 0.4) | 4 (±1.1) | 4 (± 0.8) | 0.720 |
| IPSS (1^st^ day POP^*^)  Mean (±SD) | 15.1 (± 2.6) | 14.2 (± 3.5) | 14.4 (± 1.8) | 11.5 (± 1.3) | <0.001 |
| IPSS (1^st^ week POP^*^)  Mean (±SD) | 13 (± 2.1) | 10.6 (± 2.3) | 11.9 (± 1.6) | 9.5 (± 1.4) | <0.001 |
| IPSS (3^rd^ week POP^*^)  Mean (±SD) | 13.2 (± 2.8) | 9.9 (± 3.2) | 9.8 (± 1.4) | 7.7 (± 1.3) | < 0.001 |

*POP: Postoperative day

** Comparisons are made using Anova test

**Table (4):** Comparison of stent related symptoms among all groups on 1^st^ day, 1^st^ week and 3^rd^ week postoperatively (Chinese version).

|  | **Placebo**  (Mean ± SD) | **Tamsulosin**  (Mean ± SD) | **Sofenacin**  (Mean ± SD) | **Combination**  (Mean ± SD) |
| --- | --- | --- | --- | --- |
| **1^st^ postoperative day** | 2.79 ± 0.76 | 2.48 ± 0.82 | 2.45 ± 0.55 | 1.51 ± 0.50 |
| Frequency |  |  |  |  |
| Urgency | 3.10 ± 0.85 | 2.02 ± 0.83 | 2.15 ± 0.66 | 2.02 ± 0.57 |
| Urge incontinence | 2.87 ± 0.81 | 2.31 ± 0.89 | 2.13 ± 0.68 | 1.51 ± 0.55 |
| Nocturia | 2.82 ± 0.85 | 1.85 ± 0.63 | 2.03 ± 0.70 | 1.55 ± 0.47 |
| Hematuria | 3.01 ± 0.66 | 2.02 ± 0.73 | 1.95 ± 0.59 | 1.80 ± 0.64 |
| ***Stent related pain***  Flank pain | 2.81 ± 0.72 | 2.10 ± 0.64 | 2.12 ± 0.66 | 1.97 ± 0.62 |
| Abdominal pain | 2.21 ± 0.72 | 2.41 ± 0.54 | 1.82 ± 0.63 | 1.90 ± 0.74 |
| Urethral pain | 2.48 ± 0.66 | 2.74 ± 0.63 | 2.30 ± 0.51 | 1.71 ± 0.51 |
| **1st week postoperative** | 2.01 ± 0.71 | 2.05 ± 0.60 | 2.07 ± 0.65 | 1.80 ± 0.65 |
| Frequency |  |  |  |  |
| Urgency | 3.51 ± 0.81 | 2.02 ± 0.67 | 2.28 ± 0.73 | 1.95 ± 0.59 |
| Urge incontinence | 3.00 ± 0.69 | 2.10 ± 0.64 | 2.30 ± 0.85 | 1.91 ± 0.74 |
| Nocturia | 2.82 ± 0.72 | 2.41 ± 0.54 | 2.13 ± 0.56 | 1.70 ± 0.55 |
| Hematuria | 2.92 ± 0.77 | 2.10 ± 0.64 | 2.30 ± 0.56 | 1.83 ± 0.71 |
| ***Stent related pain***  Flank pain | 2.88 ± 0.75 | 2.03 ± 0.67 | 2.23 ± 0.58 | 1.77 ± 0.57 |
| Abdominal pain | 2.91 ± 0.85 | 2.41 ± 0.55 | 1.82 ± 0.63 | 1.90 ± 0.69 |
| Urethral pain | 2.40 ± 0.71 | 2.10 ± 0.67 | 2.02 ± 0.69 | 1.75 ± 0.77 |
| **3rd week postoperative** | 3.43 ± 0.93 | 2.18 ± 0.56 | 2.37 ± 0.53 | 1.80 ± 0.66 |
| Frequency |  |  |  |  |
| Urgency | 3.23 ± 0.74 | 2.12 ± 0.67 | 2.48 ± 0.55 | 1.85 ± 0.65 |
| Urge incontinence | 3.00 ± 0.69 | 2.21 ± 0.62 | 2.13 ± 0.68 | 1.62 ± 0.54 |
| Nocturia | 2.10 ± 0.71 | 2.50 ± 0.60 | 2.47 ± 0.55 | 1.50 ± 0.55 |
| Hematuria | 2.95 ± 0.76 | 2.03 ± 0.59 | 2.32 ± 0.69 | 1.97 ± 0.62 |
| ***Stent related pain***  Flank pain | 2.10 ± 0.72 | 2.07 ± 0.63 | 2.67 ± 0.58 | 1.52 ± 0.56 |
| Abdominal pain | 2.07 ± 0.72 | 2.42 ± 0.55 | 2.21 ± 0.69 | 1.62 ± 0.54 |
| Urethral pain | 3.10 ± 0.72 | 3.07 ± 0.63 | 3.02 ± 0.69 | 2.75 ± 0.78 |
